# Spatial Patterns and Impacts of Environmental and Climatic Factors on Canine Sinonasal Aspergillosis in Northern California

**DOI:** 10.3389/fvets.2017.00104

**Published:** 2017-07-03

**Authors:** Monise Magro, Jane Sykes, Polina Vishkautsan, Beatriz Martínez-López

**Affiliations:** ^1^Center for Animal Disease Modeling and Surveillance (CADMS), Department of Medicine and Epidemiology, School of Veterinary Medicine, University of California Davis, Davis, CA, United States; ^2^William R. Pritchard Veterinary Medical Teaching Hospital (VMTH), Department of Medicine and Epidemiology, School of Veterinary Medicine, University of California Davis, Davis, CA, United States; ^3^Internal Medicine, Veterinary Specialty Center of Tucson, Tucson, AZ, United States

**Keywords:** *Aspergillus*, risk map, fungal infection, dogs, epidemiology, public health, spores, spatial analysis

## Abstract

Sinonasal aspergillosis (SNA) causes chronic nasal discharge in dogs and has a worldwide distribution, although most reports of SNA in North America originate from the western USA. SNA is mainly caused by *Aspergillus fumigatus*, a ubiquitous saprophytic filamentous fungus. Infection is thought to follow inhalation of spores. SNA is a disease of the nasal cavity and/or sinuses with variable degrees of local invasion and destruction. While some host factors appear to predispose to SNA (such as belonging to a dolichocephalic breed), environmental risk factors have been scarcely studied. Because *A. fumigatus* is also the main cause of invasive aspergillosis in humans, unraveling the distribution and the environmental and climatic risk factors for this agent in dogs would be of great benefit for public health studies, advancing understanding of both distribution and risk factors in humans. In this study, we reviewed electronic medical records of 250 dogs diagnosed with SNA between 1990 and 2014 at the University of California Davis Veterinary Medical Teaching Hospital (VMTH). A 145-mile radius catchment area around the VMTH was selected. Data were aggregated by zip code and incorporated into a multivariate logistic regression model. The logistic regression model was compared to an autologistic regression model to evaluate the effect of spatial autocorrelation. Traffic density, active composting sites, and environmental and climatic factors related with wind and temperature were significantly associated with increase in disease occurrence in dogs. Results provide valuable information about the risk factors and spatial distribution of SNA in dogs in Northern California. Our ultimate goal is to utilize the results to investigate risk-based interventions, promote awareness, and serve as a model for further studies of aspergillosis in humans.

## Introduction

Aspergillosis has gained clinical significance in both the veterinary medicine and public health fields. It affects a wide variety of hosts such as dogs, cats, birds, and humans causing morbidity and mortality worldwide (1–11). Canine sinonasal aspergillosis (SNA) is a common presentation of aspergillosis in dogs (1, 3, 12, 13) and also one of the most frequent causes of chronic sinonasal disease in dogs (6, 14–16). SNA is mainly caused by *Aspergillus fumigatus*, and less frequently by other species, such as *Aspergillus niger, Aspergillus flavus* (16, 17), and *Penicillium* spp. (16). *A. fumigatus* is a ubiquitous filamentous saprophytic fungus (18), and its conidia (spores) are found in soil, air, water, decaying vegetation, and dust. It is an airborne pathogen (19–24), thus transmission occurs through inhalation of conidia from the environment. SNA generally involves the nasal cavity and/or frontal sinuses (17, 25) of otherwise apparently systemically healthy dogs (17), causing destruction of nasal turbinates (9). Extensive damage to the nasal bones, cribriform plate, and orbit can occur in severe cases (26, 27). Clinical signs include mucoid to mucopurulent nasal discharge, sneezing, depigmentation and/or ulceration of the nares, nasal pain, and epistaxis (1, 26). Ocular discharge occurs as a result of nasolacrimal duct destruction and orbit invasion in advanced cases (1). Neurologic signs may occur if the cribriform plate is affected (26, 27). An important differential diagnosis for SNA in dogs is nasal neoplasia (14). Diagnosis is costly and requires a mixture of invasive and non-invasive diagnostic tests such as advanced imaging, serology, rhinoscopy for identification of fungal plaques, and cytology and/or histopathology (12, 27–31). Treatment is often challenging and involves a combination of tissue debridement, topical, and systemic antifungal therapy (9, 26).

We focused our study on SNA because it is a frequent form of aspergillosis seen in dogs (1, 3, 12, 13), and because the etiology and epidemiology of SNA are distinct from the other types of aspergillosis in dogs and may have implications for human disease. Nasal aspergillosis accounts for 7–34% of dogs with nasal disorders and is the second most common cause of chronic nasal discharge (6, 14, 32, 33). A study in humans shows that worldwide approximately 2.5% (4.8 million people) of adults who have asthma also have allergic bronchopulmonary aspergillosis (34). Invasive aspergillosis in humans generally affects immunocompromised people and is one of the most common fungal infections on organ transplant recipients (35). Studies have shown that *A. fumigatus* are present and propagate under a variety of environmental and climatic conditions such as water, soil, decaying vegetation, and compost. *A. fumigatus* present in the air and water for example have been associated with aspergillosis in humans (11, 19–23, 36, 37). *A. fumigatus* spores can also disperse easily in the air when compared to other types of fungi. Moisture and inappropriate temperature have been associated with the disease in birds (10).

We hypothesized that environmental and climatic factors significantly favor pathogens’ growth, spread, and disease transmission. Studies of geographical distribution with identification of potential high-risk areas and quantification of the influence of environmental and climatic factors on canine SNA are lacking.

Our specific study aims were as follows: (1) to analyze the spatial patterns of canine SNA in Northern California from electronic medical records of the University of California Davis William R. Pritchard Veterinary Medical Teaching Hospital (VMTH) to aid identification of high-risk areas for disease occurrence; (2) to assess the association between environmental/climatic risk factors and disease occurrence in Northern California using a multivariate logistic model. Although SNA does not pose zoonotic potential, dogs may be important sentinels for human exposure as dogs and humans cohabit the same environment. Therefore, this study has potential implications for both animal and public health with the ultimate goal of early detection, prevention, and mitigation of disease.

## Materials and Methods

### Data Description

The VMTH database was searched from January 1st of 1990 to December 31st of 2014 to identify cases of canine SNA. The keyword aspergi* was used to capture all canine aspergillosis cases. Dogs were then identified that had a clinical diagnosis of SNA as determined by the attending clinician. Cases were included if clinical findings were consistent with SNA based on thorough review of medical records by a board-certified internal medicine specialist (Polina Vishkautsan) including consistent medical history, physical examination findings, imaging findings (computed tomography or magnetic resonance imaging), presence of fungal plaques on rhinoscopy, positive *Aspergillus* gel immunodiffusion serology, culture of *Aspergillus* from nasal biopsy specimens and identification of fungal hyphae and conidiophores on histopathology. Not all dogs had all diagnostic tests done, but all dogs diagnosed with SNA had advanced imaging (usually a computed tomography scan) and rhinoscopy, and one or more of either visualization of fungal plaques on rhinoscopy, fungal structures on biopsy, or growth of *A. fumigatus* from a biopsy of nasal tissue. Both primary care and referral cases were included, although the majority was referred because of the need for special expertise for diagnosis and treatment of the condition.

In addition, a reference dog population was determined, which included all dogs seen at the VMTH during the 25 years of the study period residing in the zip codes within a catchment area of 145 miles around the UC Davis VMTH. This area was empirically established and assumed to represent a reasonable distance that an owner could drive to seek care. Data of cases and the dog reference hospital population were aggregated at the zip code level (i.e., unit of analysis) (Supplementary Figures S1–S3 are provided for reference).

Data collected from dogs with SNA were residential address (zip code), date of SNA diagnosis, whether diagnosis was obtained by the referring veterinarian or at the VMTH, and fungal culture results when available.

The environmental and climatic factors evaluated in this study for potential association with canine SNA occurrence are described in Table 1. We included as predictors 38 environmental and climatic factors that have a likely biological plausibility and/or have been previously described either to favor fungus growth or cause alteration or damage of respiratory track (11, 19, 22, 36–41).

**Table 1:** Environmental and climatic factors assessed for association with canine sinonasal aspergillosis occurrence over a 25-year study period.

Variable	Description (unit)	Source
Water	Open water and perennial ice/snow areas (%)	
Developed areas	Open space, low, medium, and high intensity areas of development (%)	
Barren	Barren land areas (%)	
Forest	Areas of deciduous, evergreen, and mixed forests (%)	
Shrub land	Areas dominated by shrubs (%)	Geospatial Data Getaway USDA website
Grassland/herbaceous	Areas dominated by graminoid or herbaceous vegetation (%)	National Land Cover Database 2011 by state (California)
Agriculture	Pasture/hay (areas of grasses, legumes, or grass–legume mixtures planted for livestock grazing or the production of seed or hay crops) and cultivated crops (areas used for the production of annual crops) (%)	https://gdg.sc.egov.usda.gov/
Wetlands	Woody and emergent herbaceous wetlands (%)	

Soil moisture	Mean soil moisture (average values from 1990 to 2014) (%)	Cal-Adapt website
Soil moisture difference	Difference in soil moisture (average values from 2014 minus average values from 1990) (%)	http://cal-adapt.org/data/download/
Relative humidity	Relative humidity (average values from 1990 to 2014) (%)	

Clay	Mean soil percent clay (%)	
Sand	Mean soil percent sand (%)	
Silt	Mean soil percent silt (%)	
Soil percent organic matter	Mean percentage soil organic matter (%)	Data Basin website
Soil pH	Mean soil pH (pH)	SSURGO percent soil clay, sand, silt, and pH for California, USA
Maximum soil pH	Maximum soil pH (pH)	https://databasin.org/datasets/
Minimum soil pH	Minimum soil pH (pH)	

Total land fire acre	Total acres of land at time of fire control from 1990 to 2014 (acre)	Federal Fire Occurrence website
Fire history	Counts of fire episodes from 1990 to 2014 (count)	http://wildfire.cr.usgs.gov/firehistory/data.html

Active composting sites	Active composting facilities (food, green, wood, biosolid, agricultural waste) (count)	CalRecycle website at http://www.calrecycle.ca.gov/

Wind	Mean wind speed (average values from 1990 to 2014) (m/s)	
Wind difference	Difference in wind speed (average wind speed values from 2014 minus average values from 1990) (m/s)	Cal-Adapt website
Temperature	Mean temperature (average temperature values from 1990 to 2014) (°C)	http://cal-adapt.org/data/download/
Temperature difference	Difference in temperature (average values from 2014 minus average values from 1990) (°C)	

Maximum temperature	Maximum temperature (maximum values from average maximum values from 1981 to 2010) (°F)	Prism Climate Group Oregon State University
Minimum temperature	Minimum temperature (minimum values from average minimum values from 1981 to 2010) (°F)	http://prism.oregonstate.edu/normals/
Precipitation	Mean precipitation (average values from 1981 to 2010) (″)	

Precipitation difference	Difference in precipitation (average values of 2014 minus average values of 1990) (mm)	Cal-Adapt website at http://cal-adapt.org/data/download/

Ozone	Ozone [portion of the daily maximum 8-h ozone concentration over the federal 8-h standard (0.075 ppm), averaged over 3 years (2007–2009)] (ppm)	Office of Environmental Health Hazard Assessment website http://oehha.ca.gov/ej/ces11.html
PM 2.5	Fine particulate matter annual mean concentrations (average of quarterly means), over 3 years (2007–2009) (μg/m^3^)	
Diesel PM	Diesel particulate matter emissions from on-road and non-road sources for a 2010 July day (kg/day)	
Pesticide use	Total pounds of selected active pesticide ingredients used in production-agriculture per square mile (lb/mile^2^)	
Toxic release	Total toxicity-weighted pounds of chemicals released to air or water from all facilities within the ZIP code or within 1 km of ZIP code (toxicity-weighted pounds)	
Traffic density	Sum of traffic volume (vehicle/kilometers per hour) by total road length (km) within 150 m of the ZIP code boundary [vehicle kilometers per hour/total road length (km)]	
Cleanup sites	Sum of weighted sites per ZIP codes (weighted sites)	

### Spatial Analysis

Spatial analysis was performed using *ArcGIS version 10.2.2 (ESRI^®^, 2015)* and *R Studio* (version 0.98.1091). ZIP Code Tabulation Areas (ZCTAs) shapefile for the state of California was obtained from U.S. Census Bureau (42) and joined using the *table join* function in *ArcMap* with all the information collected at the ZCTA level. The *select by location function* was used to select the ZCTA within a distance of 145 miles. We calculated the regionwide disease hospital incidence rate (i.e., sum of dog cases/sum of reference dog population) and used it to calculate the expected cases per ZCTA (sum reference dog population per ZCTA × regionwide disease incidence rate). Then, the standardized incidence ratio (SIR) was calculated by dividing the observed number of cases per ZCTA by the expected number of cases per ZCTA, and its mean 1.34 used as the cut point to create the binomial response variable for the multivariate logistic regression analysis (if the SIR value was larger than the SIR mean, it was assigned one, and if it was smaller than the mean, it was assigned 0). For the predictors, the mean value of each variable for each ZCTA was obtained using the *extract function* (*raster* package) in *R*. The *tabulate area* tool in *ArcMap* was used to obtain the percentage of land use type for each ZCTA. The geographic coordinate system used for all the shapefiles and rasters were North American 1983, the Datum North American 1983, and the projection NAD 1983 UTM Zone 11N (linear unit in meters).

### Statistical Analysis

All continuous variables were transformed and evaluated both in the standardized (*Z* = *X* − μ/σ; i.e., the observed value minus the overall mean divided by the SD) and binomial (using the median as cut point: observed value > median = 1; 0 otherwise) forms. Univariate logistic regression analysis was first applied to each variable and only those that had a *p*-value smaller than 0.25 were considered for inclusion in the full model. Then, a multivariate logistic regression analysis was used to identify the factors significantly associated with the disease occurrence. The model was specified as follows:

$$\text{logit}(p)=\text{ln}\left(\frac{p}{1-p}\right)=\alpha +\beta_{1}\chi_{1}+\beta_{2}\chi_{2}\ldots +\beta_{k}\chi_{k}$$

where *p_i_* is the probability of SNA occurrence at ZCTA level, α is the intercept and, β are the regression coefficients for each predictor χ.

Model selection was conducted using forward selection (*step function* from *stats package* from *R*) and using the *Akaike information criterion* to determine the best fitting model. Model diagnostics were verified by checking deviance residuals and the variance inflation factor to evaluate multicollinearity problems. The predictive ability of the model was conducted analyzing the area under the curve (AUC) of the receiver operating characteristic (ROC) curve. All possible two-way interaction terms and potential confounders were also evaluated. Fitted values obtained were then used to create a canine SNA risk map using the *spplot function* (*sp* package) in *R*. The *spplot function* was also used to create a deviance residuals map obtained from the logistic regression model to analyze the distribution and presence of extreme residual values. Because the neglect of spatial autocorrelation can result in biased regression coefficient estimates for the SNA occurrence, an autologistic regression analysis was run and compared with the ordinary multivariate logistic regression analysis results and check for any effect on spatial autocorrelation. The autologistic regression model introduced by Besag (43) is an extension of an ordinary logistic regression model and corrects for spatial dependence in the observations by incorporating an autocovariate variable. In this case, the autocovariate variable was generated from a neighborhood-weighting matrix based on the probabilities of SNA occurrence obtained from the final multivariate logistic regression model. Among the several spatial weights tested (i.e., rook and queen contiguity, inverse-distance) to create the spatial weights matrix, queen contiguity was considered the most appropriate for this neighborhood structure and was chosen. Queen contiguity defines ZCTAs neighbors when they either share a border or vertex. The autocovariate formula was specified as follows:

$$\text{Auto }{\text{cov}}_{i}=\frac{\sum_{j=1}^{k_{i}}w_{ij}{\hat{p}}_{J}}{\sum_{j=1}^{k_{i}}w_{ij}}$$

The autocovariate variable (*Auto cov_i_*) is a weighted average of the probabilities of the geographic units (ZCTAs) amongst a set of *k_i_* neighbors of the geographic unit *i, w_ij_* is the spatial weight between the geographic unit *i* and *j*, $\hat{p_{j}}$ is the probability estimated by the logistic regression model. All analyses were conducted in *R* Language using *R Studio* (44, 45).

## Results

A total of 250 cases (out of 286 cases retrieved from ZCTA in the entire state of California) and 190,894 dogs (reference dog population) were part of the study catchment area ZCTAs and were considered for the analysis. The mean number of cases per ZCTA in the study area was 0.33 (SD = 0.71; min = 0; max = 5). The overall cumulative incidence (incidence proportion) for the study area was 1.31 SNA cases per 1,000 dogs for the entire studied period. The mean SIR was 1.34 (SD = 4.96; min = 0; max = 54.54). The study area contained 768 ZCTAs (43% of the total number of ZCTAs in California), from which 140 ZCTAs had an SIR > mean.

The final model contained six variables (five main effects and one interaction term), three of which were environmental [i.e., traffic density (OR = 1.7; *p* = 0.0311), active composting sites (OR = 1.2; *p* = 0.0299), agriculture (OR = 0.67; *p* = 0.00345)] and three were climatic [i.e., wind difference (OR = 1.3; *p* = 0.0621), temperature difference (OR = 0.69; *p* = 0.0104), interaction between wind and temperature differences (OR = 1.6; *p* = 0.0134)] (Table 2). The predictive ability of the model based on the AUC value of the ROC curve was 73.1%. The autologistic regression model showed similar results than the final logistic regression model, with similar regression coefficients, deviance residuals and AUC and, for that reason, the simplest logistic regression model was selected.

**Table 2:** Association between environmental and climatic variables and canine sinonasal aspergillosis occurrence in California obtained with a multivariate logistic regression model.

Variable	Odds ratio (95% confidence interval)	*p*-Value (Wald test)
Traffic density^a^ low (≤503.2)	Reference = 1.0	
High (>503.2)	1.7 (1.1, 2.8)	0.0311
Wind difference (2014–1990)^b^	1.3 (0.99, 1.7)	0.0621
Active composting sites^b^	1.2 (1.0, 1.4)	0.0299
Temperature difference (2014–1990)^b^	0.69 (0.52, 0.92)	0.0104
Agriculture^b^	0.67 (0.52, 0.88)	0.00345
Wind difference × temperature difference^b^	1.6 (1.1, 2.3)	0.0134

*^a^Binomial form*.

*^b^Standardized form*.

**Figure 1:**
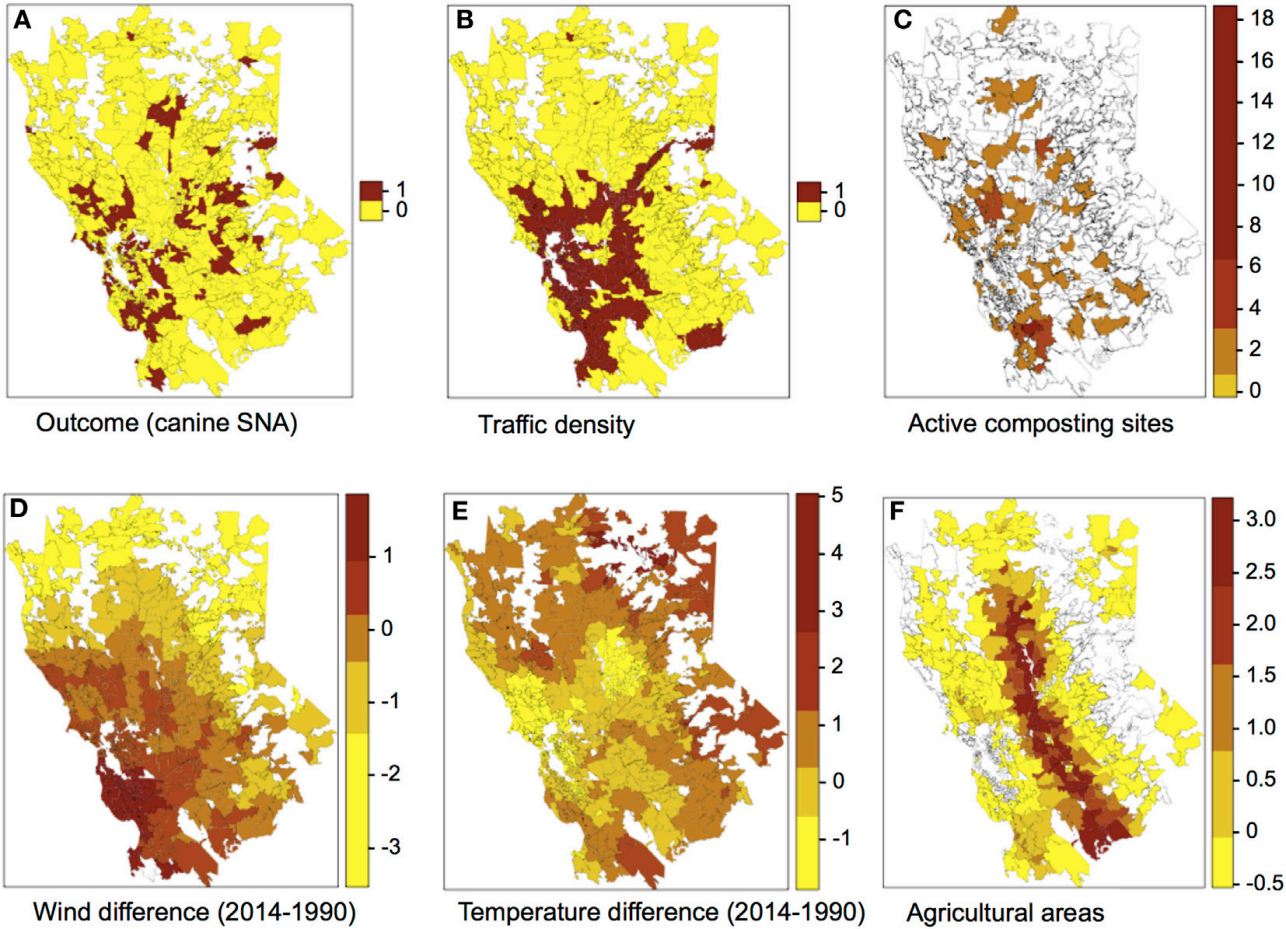
Spatial distribution of the outcome **(A)** and predictors **(B–F)** included in the final multivariate logistic regression model. Categories for the colors of plots **(C–F)** were obtained using the Jenks algorithm (i.e., Natural breaks).

**Figure 2:**
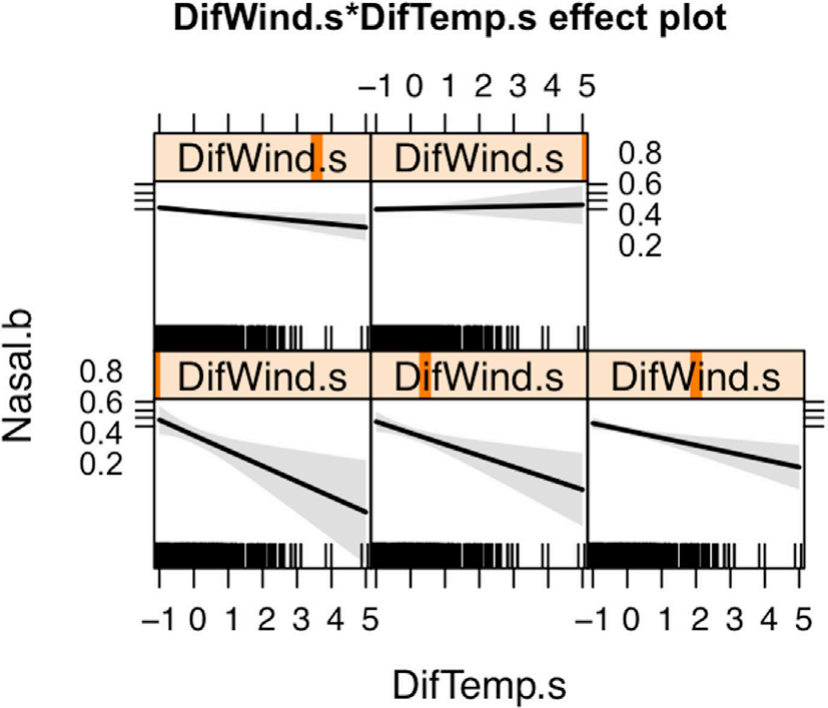
Effect of the interaction between wind and temperature differences on the occurrence of canine sinonasal aspergillosis in Northern California. Vertical orange lines indicate the value of the wind difference considered for each plot.

The spatial distribution of SNA and significant predictors included in the final model are shown in Figure 1. The effect of temperature difference interacting with wind difference at different levels in the occurrence of SNA is shown in Figure 2. This figure shows that when there was high wind difference, the probability of SNA was high, and the protective effect of temperature difference on SNA occurrence was minimized.

**Figure 3:**
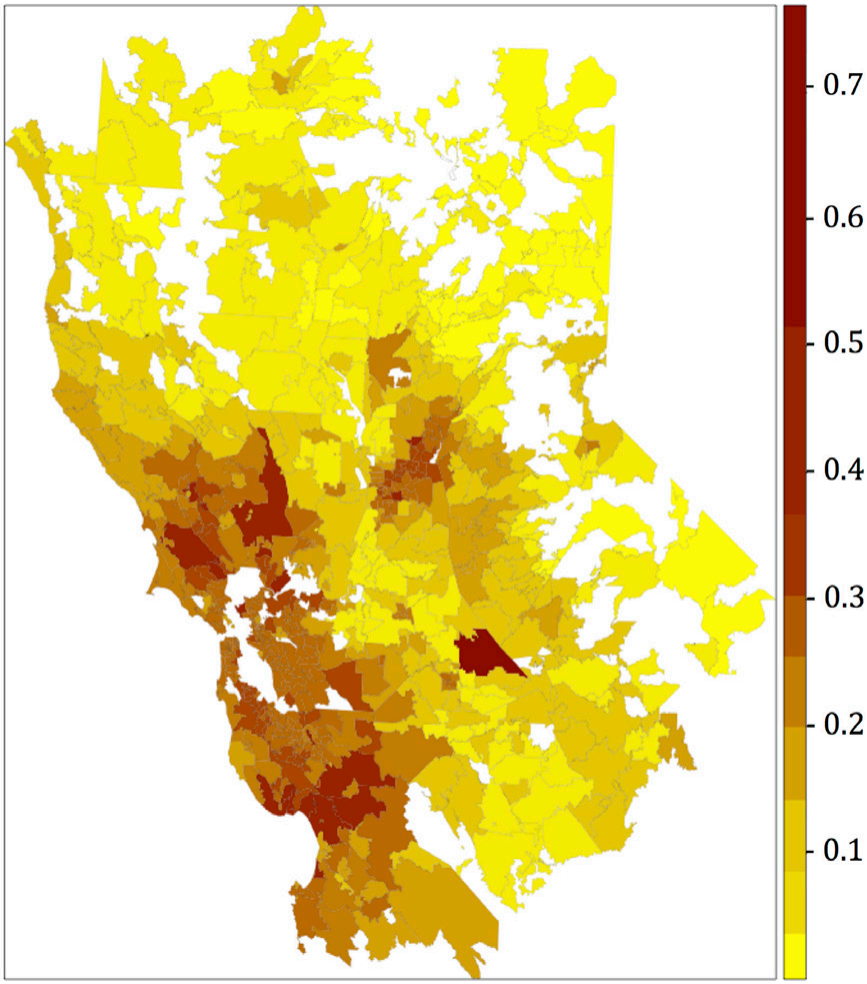
Canine sinonasal aspergillosis risk map obtained after plotting the fitted values of the final multivariate logistic regression model. Categories for the colors were obtained using the Jenks algorithm (i.e., natural breaks).

**Figure 4:**
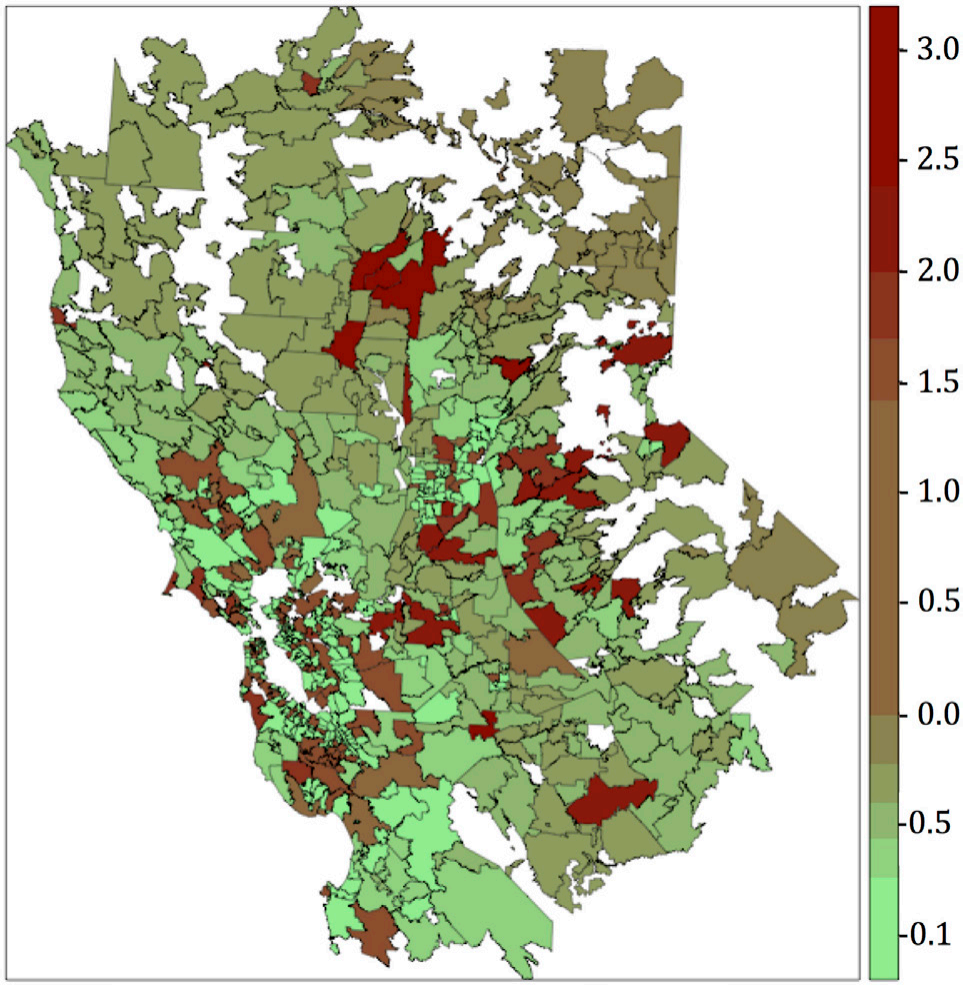
Residuals map obtained after plotting the deviance residuals of the final multivariate logistic regression model. Categories for the colors were obtained using the Jenks algorithm (i.e., natural breaks).

The resultant risk map for canine SNA shows that high-risk areas were concentrated close to the San Francisco Bay area, other coastal areas, and also in northern and central CA (Figure 3). The top 10 counties containing ZCTAs that exhibited the highest risk for canine SNA occurrence were Stanislaus, Santa Clara, Sonoma, Santa Cruz, Napa, Contra Costa, Placer, Monterey, Marin, and Sacramento. Residuals from the final logistic model were relatively randomly distributed within the study area (Figure 4).

## Discussion

To the best of authors’ knowledge, this is one of the first studies to provide a canine SNA risk map and to analyze the association between environmental and climatic factors associated with canine SNA in California. Results provide valuable insights on climatic, environmental, and anthropogenic risk factors associated with the disease.

We compared results from both models (logistic and autologistic) by evaluating improvements in the intercept, regression coefficients, deviance residuals, and goodness of the fit (AUC). Since the autologistic model did not significantly change the results over the logistic model, we assumed that spatial autocorrelation was not playing an important role in this particular case. Moreover, residuals map for the final logistic model did not show values too far from 0, and the highest values were fairly dispersed. The majority of the regions with the highest deviance residual values (residual map values >2) are located mainly in the sierra and do not overlap with the highest risk areas (risk map values >0.4), located mainly in the coastal areas. Overall, the deviance residual values are low, indicating a good fit of the model, and are generally dispersed, which, in addition to the results of the autologistic regression model suggest no concerning spatial autocorrelation. Only values deviance residuals >2 may be indicative of lack of fit for those few counties in the sierra suggesting that our model seems to predict better coastal cases than cases in the sierra. Thus, the non-spatial logistic regression model was chosen for simplicity. Traffic density was the main factor associated with SNA (OR = 1.7), suggesting that pollution may predispose dogs to this disease. It is possible that the pathogenicity of *A. fumigatus* in dogs varies in the presence of different pollutants, or that pollution damages mucosal defense mechanisms. One recent study of the effect of urban air pollution on allergenicity of *A. fumigatus* spores in the laboratory found increased allergenicity in polluted urban environments during the first 12 h of exposure (46).

Wind and temperature were also significantly associated with SNA occurrence. Both wind and temperature differences between the beginning and the end of the study period (i.e., 1990 and 2014, respectively) were used as proxies of areas where climatic conditions are more drastically changing. Both wind and temperature measurements were higher in 2014 compared to 1990. The protective effect that temperature difference had on SNA occurrence was attenuated by wind (Figure 2). Wind may facilitate dispersion of spores and thus increase the risk of SNA in dogs. Areas with increased wind in the last years are mostly in urban and coastal settings, which tend to experience landscape modifications such as construction, leading to soil disturbance. Urban and coastal areas also appear to have higher humidity and traffic density compared to other areas. Similarly, this study suggests that dogs living in areas experiencing lower temperature differences are those at highest risk of SNA, although again this effect was influenced by wind. In general, these areas have lower elevations and higher temperatures (Figure 1). Active composting sites (for food, green waste, wood, biosolid, and agricultural waste) were significantly associated with an increased risk for SNA. *A. fumigatus* thrives in composting facilities due to the presence of organic matter (22, 37, 39). The thermotolerant nature of *A. fumigatus* allows this pathogen to grow in compost and crops at elevated temperatures (optimal growth at 37°C and maximum growth at 52°C) (47, 48).

Agricultural land use by ZCTA reduced the risk of canine SNA. This was somewhat unexpected because the fungus is frequently found in environments rich in nutrients and humidity and where the soil is usually disturbed (as it is usually the case of agricultural areas). However, one possible explanation for the negative association is fungicide application to crops. Unfortunately, detailed information about fungicide use in California was not available. Further studies should be conducted to clarify this effect.

Risk maps provide useful information for clinicians when evaluating animals with chronic nasal discharge that reside in high-risk areas and may help to accelerate accurate diagnosis, and in turn enable more prompt treatment and better prognosis. Furthermore, because *A. fumigatus* causes disease in both dogs and humans, and because dogs and humans share the same environments, our findings may also have relevance for identification of high-risk areas for human aspergillosis. Human invasive aspergillosis mostly affects immunocompromised patients and can be life-threatening (4, 38, 49, 50). Because *A. fumigatus* spores can be found in air, dust, and water in hospital environments (11, 20, 36, 38, 41), hospitals located in high-risk areas might focus on improved ventilation systems to reduce risk of infection.

One of the limitations of this study was the availability of data from a single hospital, which can introduce selection bias. Because canine SNA requires specialized diagnostic equipment and treatments not commonly available in general veterinary practice, a diagnosis of canine SNA is often made at the VMTH or cases are referred in for treatment. In order to further reduce selection bias, the 145-mile catchment area around the VMTH was empirically delineated to represent areas of hospital caseload influence. This radius was assumed to be a reasonable distance for a pet owner to drive to seek treatment at the VMTH. Thirdly, the dog reference hospital population from the 25-year study period was taken into account for the SIR calculation; therefore, we adjusted the expected number of SNA cases observed by ZCTA with the population of dogs visiting VMTH from that ZCTA. Therefore, the use of SIR provides a better estimate of the SNA situation per region than the use of the number of cases *per se*. Lack of access to patient travel history was also a limitation of this study since canine SNA is a chronic disease, and some dogs may have become infected at a different residence prior to diagnosis. Similarly, a long study period was used because of the relatively low number of cases/year, and the relatively stable SIR over that 25-year period (mean: 1.34; SD: ±4.96). The variability of 4.96 is not very high, but it is important to consider. We tried to apply Bayesian zero-inflated Binomial and zero-inflated Poisson models that allow for overdispersion in INLA to model these data, but models did not converge. Authors acknowledge the limitation of merging cases over time and unfortunately it was not possible to include variables per year to minimize ecological fallacy due to the small sample size. However, changes over time were verified and variables such as difference in temperature, in wind, and in precipitation were included and used to account for changes over time and hopefully mitigate the impact of time on the study results.

Future studies that include data from more veterinary hospitals over a wider geographic range may provide more detail in regards to the spatiotemporal patterns of this disease and the varying impact that environmental and climatic conditions may have had in different regions over time.

Despite the limitations, this study provides preliminary insights into the spatial distribution and risk factors contributing to SNA occurrence in dogs in Northern California. These results may be useful to increase awareness and guide diagnosis and risk mitigation strategies in high-risk areas, ultimately opening new doors for further investigation of *A. fumigatus* infections not only in dogs, but also in humans.

## Author Contributions

All authors contributed to the design of the study. PV, JS, and MM contributed to the data gathering, cleaning, interpretation, and validation. MM and BM-L wrote the R codes, conducted the data analyses, and drafted the manuscript. All the authors contributed to the critical discussion of results and reviewed and edited the final manuscript.

## Conflict of Interest Statement

The authors declare that the research was conducted in the absence of any commercial or financial relationships that could be construed as a potential conflict of interest.
