# Supplementary material for: Spatial Patterns and Impacts of Environmental and Climatic Factors on Canine Sinonasal Aspergillosis in Northern California
Source: Front Vet Sci. 2017 Jul 3;4:104. doi: 10.3389/fvets.2017.00104 (PMC5494614; doi:10.3389/fvets.2017.00104)
Supplement: Supplementary file 1 [file Data_Sheet_1.DOCX]

**Supplementary Material:**


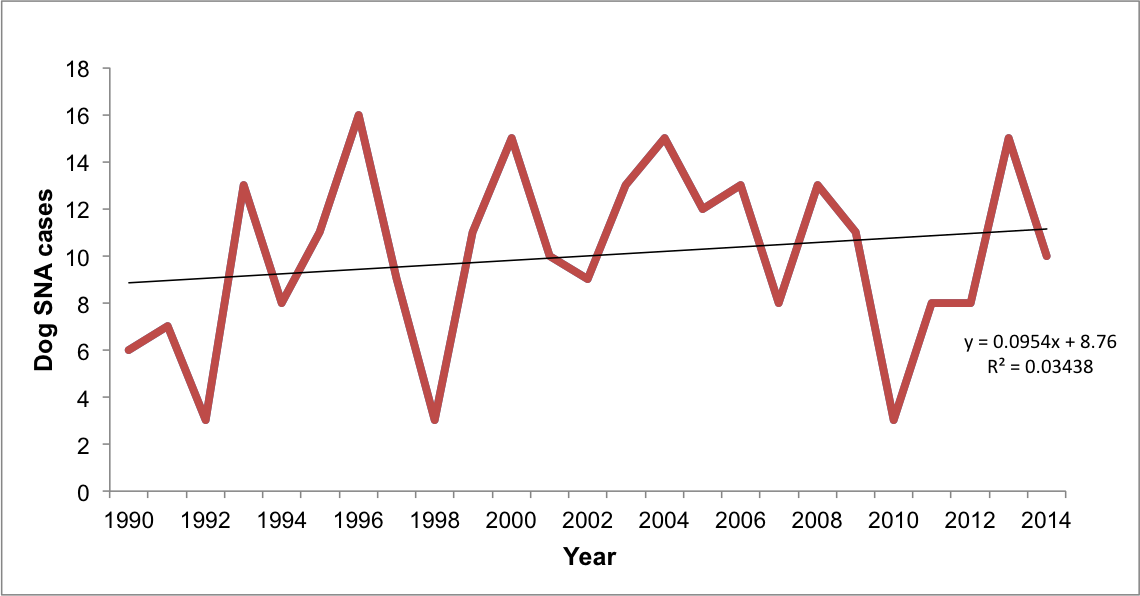


Figure 1. Dog SNA cases by year (1990-2014).


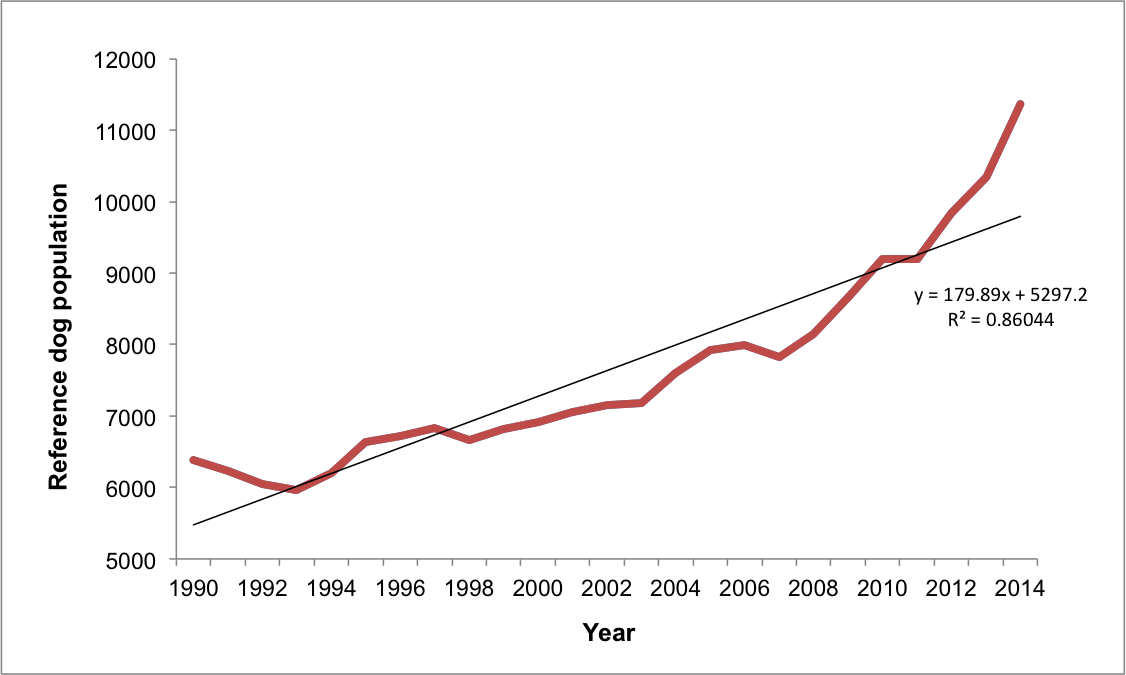


Figure 2. VMTH reference dog population by year (1990-2014).


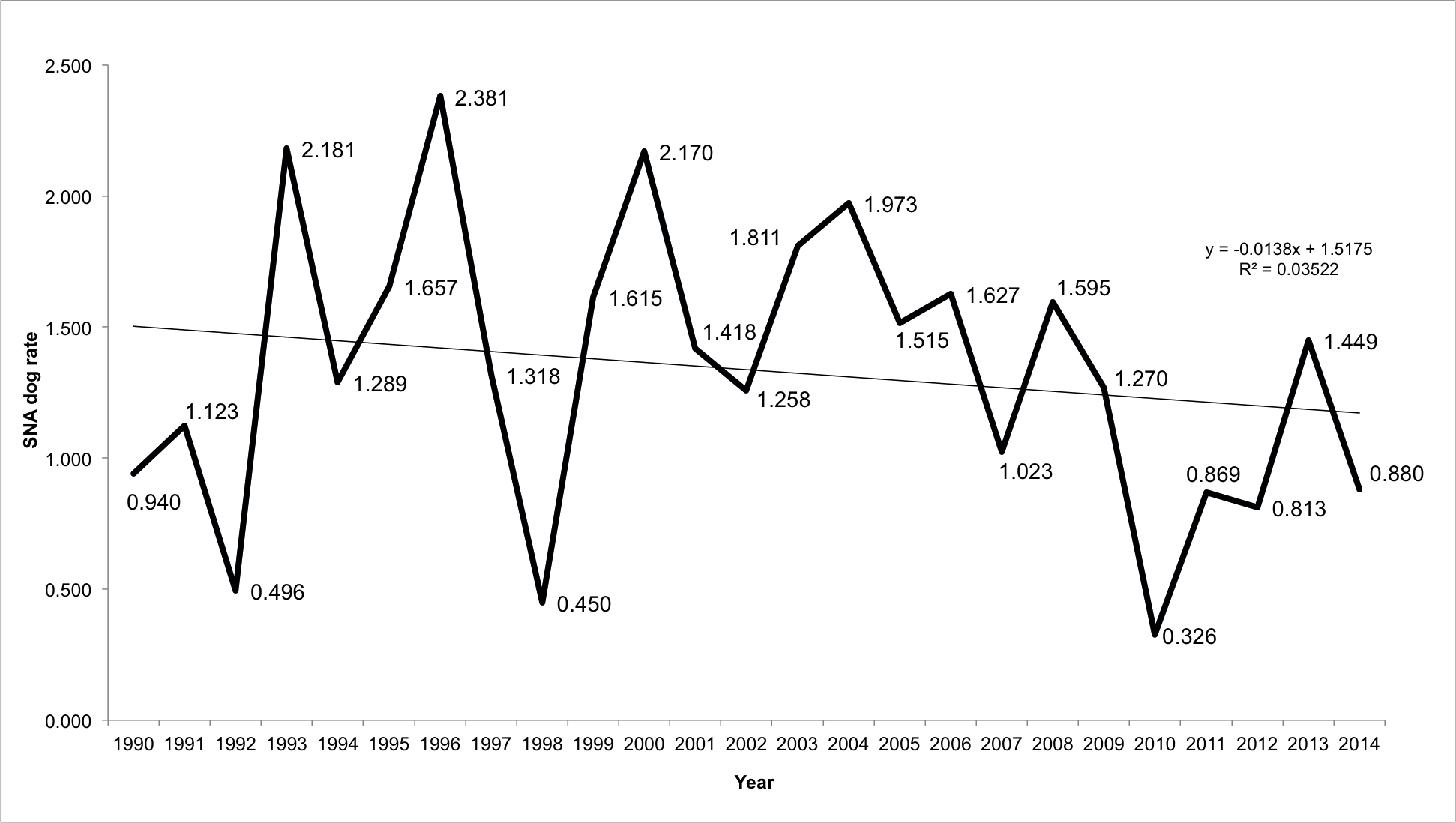


Figure 3. SNA dog rate (SNA cases/Reference Dog Population per 1000 dogs) by year (1990-2014).
